# Analysis of Phenolic Compounds and Antioxidant Activity in Wild Blackberry Fruits

**DOI:** 10.3390/ijms160714540

**Published:** 2015-06-26

**Authors:** Jan Oszmiański, Paulina Nowicka, Mirosława Teleszko, Aneta Wojdyło, Tomasz Cebulak, Krzysztof Oklejewicz

**Affiliations:** 1 Department of Fruit and Vegetable Processing, Wroclaw University of Environmental and Life Sciences, Wroclaw 51-630, Poland; E-Mails: paulina.nowicka@up.wroc.pl (P.N.); miroslawa.teleszko@up.wroc.pl (M.T.); aneta.wojdylo@up.wroc.pl (A.W.); 2 Faculty of Biology and Agriculture, University of Rzeszów, Rzeszów 35-601, Poland; E-Mails: tomcel@univ.rzeszow.pl (T.C.); koklej@univ.rzeszow.pl (K.O.)

**Keywords:** wild blackberry, phenolic compounds, extraction methods, pressurized liquid extraction, ultrasound-assisted extraction

## Abstract

Twenty three different wild blackberry fruit samples were assessed regarding their phenolic profiles and contents (by LC/MS quadrupole time-of-flight (QTOF) and antioxidant activity (ferric reducing ability of plasma (FRAP) and 2,2-azinobis (3-ethyl-benzothiazoline-6-sulfonic acid) (ABTS)) by two different extraction methods. Thirty four phenolic compounds were detected (8 anthocyanins, 15 flavonols, 3 hydroxycinnamic acids, 6 ellagic acid derivatives and 2 flavones). In samples, where pressurized liquid extraction (PLE) was used for extraction, a greater increase in yields of phenolic compounds was observed, especially in ellagic acid derivatives (max. 59%), flavonols (max. 44%) and anthocyanins (max. 29%), than after extraction by the ultrasonic technique extraction (UAE) method. The content of phenolic compounds was significantly correlated with the antioxidant activity of the analyzed samples. Principal component analysis (PCA) revealed that the PLE method was more suitable for the quantitative extraction of flavonols, while the UAE method was for hydroxycinnamic acids.

## 1. Introduction

The subgenus *Rubus* (blackberry) is composed of numerous, difficult to identify species found throughout the Northern Hemisphere. Blackberry chemical composition varies on the basis of variety, growing conditions, stage of ripeness and harvest and storage conditions [1]. In fact, blackberries’ medicinal qualities have been known since the 16th century in Europe, where they were used to treat infections of the mouth and eyes. Blackberry is a fruit of interest because of its high content of anthocyanins and ellagitannins, as well as other phenolic compounds that contribute to its high antioxidant capacity [2]. These fruits have high antiproliferative and anti-inflammatory properties [3]. Blackberries are currently promoted as a rich source of polyphenols, compounds of interest because of their antioxidant activity as radical scavengers, and their possible beneficial roles in human health, such as reducing the risk of cancer, cardiovascular disease and other pathologies [4,5,6,7].

Phenolic compounds were analyzed in blackberry fruits, but the amounts reported were closely dependent on the analytical conditions [8]. A great deal of attention is being paid to the extraction mechanisms used for the analysis of phenolic compounds and antioxidant activity in blackberry fruits. After sample preparation, the complete extraction of phenolic compounds is the critical step. The most common techniques for extracting phenolics employ solvents, either organic or inorganic. Several parameters may influence the yield of phenolics, including extraction time, temperature, solvent-to-sample ratio, number of repeated extractions of the sample and solvent type. Consequently, the optimum recovery of phenolics is different from one sample to the other and relies on the type of plant and its active compounds [9,10].

The most commonly-used methods for compound extraction are conventional liquid-liquid or solid-liquid extraction. The uses of pressurized liquid extraction (PLE) and ultrasound-assisted extraction (UAE) are increasing [11]. These methods shorten extraction times, decrease the release of toxic pollutants through reducing organic solvent consumption and are relatively simple to perform [9]. PLE is referred to as an accelerated, automated technique using solvents for the extraction of phenolics from plant material. PLE operates with nitrogen at high temperature and pressure, which helps the solvent penetrate rapidly into the plant cells and prevents the degradation of phenolic compounds. The method uses organic liquid solvents at high temperature (50 to 200 °C) and pressure (1450 to 2175 psi) to ensure the rapid extraction rate of compounds [10]. Thus, temperature could be used to match the polarity of a solvent to that of the compounds of interest to be recovered [9,10]. The high pressure helps the extraction cells to be filled faster and forces liquid into the solid matrix [11].

The extraction of phenolic compounds by PLE has been investigated in numerous studies, which have presented several approaches to optimizing extraction conditions or evaluating their efficiency compared with other methods [12,13,14,15].

A wide variety of solvents may be employed in the PLE method, and the most often used are water and methanol or ethanol [16]. PLE was used as a sustainable, green extraction technique, while functional characterization was carried out by using different *in vitro* assays, including total phenolic determination, as well as two different antioxidant capacity assays, 2,2-diphenyl-1-picrylhydrazyl (DPPH) and trolox equivalent antioxidant capacity (TEAC). Moreover, extracts were chemically characterized by using the LC–MS/MS method to correlate antioxidant activities with a particular chemical composition.

Other solvent extraction methods, such as ultrasound-assisted extraction (UAE), are also used for the extraction of phenolic compounds [17]. The use of ultrasound (sound waves that have frequencies higher than 20 kHz) can disrupt plant cell walls with a subsequent increase in solvent penetration, which helps in obtaining a higher extraction yield. Sonication is the production of sound waves that create cavitation bubbles near the sample tissue, which break down to disrupt cell walls, thereby releasing cell contents. Ultrasound-assisted extraction can be a technique of choice for thermolabile components, as the operating temperature can remain low during this process, thus maintaining extract quality [18]. Compared with conventional methods, UAE is one of the most simple, inexpensive extraction systems. The application of ultrasound helps the extraction yield and speeds up the extraction rate in solid-liquid extraction. Ultrasounds have been applied to the extraction of phenolics from different plants [18,19,20,21]. However, Biesaga [22] has shown that phenolic compounds extracted by UAE were degraded, and the effect of the degradation of flavonoids depends on the extraction mode and chemical structure.

So far, to our knowledge, there have been no comparative studies on the chemical composition of berries of a large number of *Rubus* species. Consequently, the purpose of this study was to identify a broad range of phenolic acids and flavonoids and their contents in berries of 23 species belonging to the *Rubus* genus and to compare them. This is the first paper about flavonoids and the phenolic acid composition of numerous members of the multispecies *Rubus* genus. Additionally, in the present study, the relationship between extraction yields of phenolic compounds (anthocyanins, phenolic acids, flavonols and ellagic tannins) and antioxidant activity under different extraction techniques using PLE and UAE for 23 wild blackberry fruit sample was also discussed.

## 2. Results and Discussion

### 2.1. Peak Identification and Assignment

**Figure 1:**
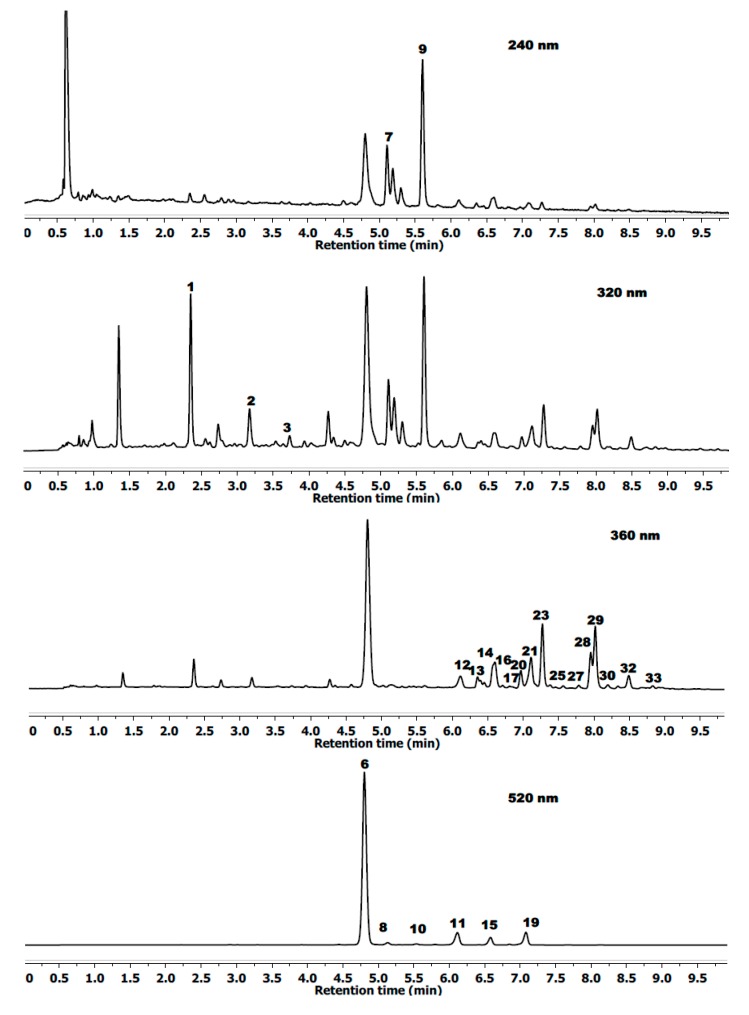
Example of a typical chromatographic profile of the main phenolic compounds from *Rubus radula* at 260, 320, 360 and 520 nm. For the peak labels, see Table 1.

Identification and peak assignment of phenolic compounds in blackberry fruits were based on a comparison of their retention times and mass spectral data with those of standards and additionally with published data (Table 1; Figure 1). Thirty four phenolic compounds were detected in wild blackberry fruit. Eight of them were cyanidin derivatives (MS/MS ion at *m*/*z* 287.0571). From a comparison of its mass spectral data with those reported previously, these anthocyanins were tentatively identified as cyanidin-3-diglucoside, -3-glucosyl-rutinoside, -3-glucoside, -3-rutinoside, -3-(3ʹ-malonoyl)-glucoside, -3-xyloside, -3-(6ʹ-malonyl)-glucoside and -3-dioxaloylglucoside [11,23,24].

Blackberries have a complex flavonol profile due to the occurrence of ten quercetin (MS/MS ion at *m*/*z* 301.0277) and five kaempferol (MS/MS ion at *m*/*z* 285.0187) derivatives (Table 1). In analyzed blackberry fruit extracts, monoglucoside as two quercetin-3-*O*-pentoside (MS ion at *m*/*z* 433.0777), quercetin-3-*O*-rhamnoside (MS ion at *m*/*z* 447.0968), three quercetin-3-*O*-hexoside (MS ion at *m*/*z* 463.0843) and quercetin-3-*O*-glucuronide (MS ion at *m*/*z* 477.0968) were found. Other quercetin derivatives, such as 3-methoxyhexoside (MS ion at *m*/*z* 493.1001), -3-*O*-rutinoside (MS ion at *m*/*z* 609.1080) and -3-(6ʹ-(3-hydroxy-3-methylglutaroyl)-galactoside (MS ion at *m*/*z* 607.1293) were identified according to previously published data [2,6,7,24,25].

**Table 1:** The characterization of phenolic compounds of blackberry fruits using their spectral characteristics in ultra-pressure liquid chromatography with photodiode array and mass spectrometry (UPLC-PDA/MS) by retention time, λ_max_ and negative and positive ions.

Compounds ^‡^	Rt	λ_max_	(MS)^−^	(MS/MS)^−^
	(min)	(nm)	(*m*/*z*)	(*m*/*z*)
Chlorogenic acid	2.35	323	353.0866	235.9249/191.0553/146.9378
Caffeoyl hexoside	3.14	320	341.0849	179.0349/135.0464
*p*-Coumaric acid	3.69	312	163.0380	
Cyanidin-3-*O*-diglucoside	4.21	514	611.1664^+^^†^	287.0571^+^
Cyanidin-3-glucosylrutinoside	4.36	517	757.2241^+^	611.1513/449.1063/287.0571^+^
Cyanidin-3-*O*-glucoside	4.74	514	449.1063^+^	287.0571^+^
Ellagitannins Lambertianin C	5.00	244	1401.3730	633.075/300.9999
Cyanidin-3-*O*-rutinoside	5.08	516	595.1664^+^	287.0571^+^
Ellagitannins hex (casuarinin)	5.51	244	935.0760	633.075/300.9999
Cyanidin-3-(3ʹ-malonyl)glucoside	5.74	515	535.1084^+^	287.0571^+^
Cyanidin-3-*O*-xyloside	6.08	514	419.0987^+^	287.0571^+^
Ellagic acid pentoside	6.28	360	433.0777	300.9999
Quercetin-3-methoxyhexoside	6.38	360	493.1001	463.3010
Ellagic acid	6.51	364	300.9999	
Cyanidin-3-(6ʹ-malonyl)glucoside	6.54	517	535.1084^+^	287.0571^+^
Ellagic acid rhamnoside	6.64	360	447.0527	300.9999
Kaempferol-3-*O*-glucoside-rhamnoside-7-*O*-rhamnoside	6.73	346	739.1930	593.1559/285.0187
Quercetin-3-*O*-rutinoside	6.90	352	609.1080	463.0397/301.0277/151.0034
Cyanidin-3-dioxalylglucoside	7.03	517	593.1520^+^	287.0571
Quercetin-3-*O*-galactoside	7.04	353	463.0843	301.0277/151.0034
Quercetin-3-*O*-glucuronide	7.14	351	477.0670	301.0277/151.0034
Quercetin-3-*O*-glucoside	7.20	352	463.0843	301.0277/151.0034
Kaempferol derivative	7.27	345	475.0125	447.0968/285.0187
Quercetin-3-*O*-hexoside	7.36	352	463.0843	301.0277/151.0034
Kaempferol-3-*O*-rutinoside	7.48	350	593.1559	447.0968/285.0187
Luteolin-3-*O*-glucuronide	7.59	340	461.0710	285.0187
Quercetin-3-*O*-pentoside	7.88	352	433.0777	301.0277/151.0034
Quercetin-3-(6ʹ-(3-hydroxy-3-methylglutaroyl)-galactoside	7.94	345	607.1293	463.0843/301.0277/151.0034
Quercetin-3-*O*-pentoside	8.12	352	433.0777	301.0277/151.0034
Quercetin-3-*O*-rhamnoside	8.28	350	447.0968	301.0277/151.0034
Kaempferol-3-*O*-glucuronide	8.43	346	461.0710	285.0187
Methyl ellagic acid pentose	8.60	360	477.1082	314.0421/300.9996
Kaempferol-3-*O*-pentoside	8.76	350	417.0397	285.0187
Apigenin-3-*O*-glucuronide	8.90	338	445.0710	269.0450

^†^ Molecular ion [M + H]^+^; ^‡^ All compounds were identified in all analyzed species, but at different levels.

Five kaempferol derivatives were identified as -*O*-glucoside-rhamnoside-7-*O*-rhamnoside with *m*/*z* 739.1930 and an MS/MS fragment at 593.1559 obtained after the loss of 146 amu (rhamnose moiety) and MS/MS fragments at 285.0187 after the loss of 308 amu (rhamnoglucoside moiety), -*O*-rutinoside (MS ion at *m*/*z* 593.1559), -*O*-glucuronide (MS ion at m/z 461.0710), -*O*-pentoside (MS ion at *m*/*z* 417.0397) and non-identified kaempferol derivative (MS ion at *m*/*z* 475.0271 with fragmentation MS/MS ion at *m*/*z* 447.0968 and 285.0187). Cho *et al.* [2] reported the presence of these kaempferol derivatives in blackberry fruits.

Two flavones were detected in the wild blackberry fruit extracts: luteolin-3-*O*-glucuronide (MS ion at *m*/*z* 461.0710 with fragmentation MS/MS ion at *m*/*z* 285.0187) and apigenin-3-*O*-glucuronide (MS ion at *m*/*z* 445.0710 with fragmentation MS/MS ion at *m*/*z* 269.0450). These compounds had maximum absorption at shorter wavelengths (340 and 338 nm) than flavonols, which indicated their presence in analyzed samples.

Three hydroxycinnamic acid derivatives were detected in the blackberry fruit extracts. Among them were chlorogenic acid and *p*-coumaric acid identified by comparison with standard compounds and caffeoyl hexoside with *m*/*z* 341.0849 and an MS/MS fragment at 179.0349 obtained after the loss of 162 amu (hexose moiety).

Blackberry fruits are rich sources of ellagitannin and ellagic acid derivatives [26,27]. In the biosynthetic pathways of plants, gallotannins are transformed to ellagitannins by oxidative C–C coupling between two spatially-adjacent galloyl groups, and hexahydroxydiphenoyl (HHDP) groups are formed. Therefore, ellagitannins exhibit shorter wavelengths for absorption maxima and weaker absorption intensities in the region of 275 to 285 nm than galloyl esters (including gallotannins), depending on the number of HHDP and galloyl groups present in the molecules [28]. Ellagic acid (MS ion at *m*/*z* 300.9999) and ellagic acid pentoside (MS ion at *m*/*z* 433.0777), rhamnoside (MS ion at *m*/*z* 447.0527) and methyl ellagic acid pentose (MS ion at *m*/*z* 477.1082) were identified in blackberry extracts based on mass spectral data and a comparison of their retention times (ellagic acid) with those of standards and published data (Table 2) [27,28,29,30]. Two ellagitannins, Lambertianin C (MS ion at *m*/*z* 1401.3730) and ellagitannins hexoside (casuarinin) (MS ion at *m*/*z* 935.0760), were identified in the wild blackberry fruit extracts based on maximum absorption at 240 nm, mass fragmentation spectral data *m*/*z* 633.0750 (galloyl-HHDP glucose) and *m*/*z* 300.9999 (ellagic acid), and published data [27,31].

**Table 2:** Sample numbers and areas of wild blackberry fruit harvesting.

Number of Sample	Blackberry Species	Geographical Location	
1	*Rubus radula*	Albigowa Honie	N 50°0ʹ19.28ʹʹ E 22°10ʹ22.06ʹʹ
2	*Rubus montanus*	Berendowice	N 49°40ʹ14.85ʹʹ E 22°43ʹ39.58ʹʹ
3	*Rubus gracilis*	Las Niechciałka	N 50°5ʹ45.38ʹʹ E 22°35ʹ45.06ʹʹ
4	*Rubus macrophyllus*	Las Niechciałka	N 50°5ʹ45.38ʹʹ E 22°35ʹ45.06ʹʹ
5	*Rubus pericrispatus*	Kopystno	N 49°41ʹ8.38ʹʹ E 22°38ʹ32.49ʹʹ
6	*Rubus austoslovacus*	Długie k/Przemyśla	N 49°45ʹ49.61ʹʹ E 22°42ʹ4.59ʹʹ
7	*Rubus subcatus*	Łazy k/Birczy	N 49°42ʹ49.56ʹʹ E 22°32ʹ3.14ʹʹ
8	*Rubus ambrosius*	Zmysłówka	N 50°9ʹ58.91ʹʹ E 22°22ʹ43.39ʹʹ
9	*Rubus fasciculatus*	Łazy k/Birczy	N 49°42ʹ49.56ʹʹ E 22°32ʹ3.14ʹʹ
10	R*ubus nessersis*	Las Niechciałka	N 50°5ʹ45.38ʹʹ E 22°35ʹ45.06ʹʹ
11	*Rubus glivicensis*	Zmysłówka	N 50°9ʹ58.91ʹʹ E 22°22ʹ43.39ʹʹ
12	*Rubus caesius*	Długie k/Przemyśla	N 49°45ʹ49.61ʹʹ E 22°42ʹ4.59ʹʹ
13	*Rubus bifronus*	Berendowice	N 49°40ʹ26.44ʹʹ E 22°43ʹ6.76ʹʹ
14	*Rubus praecox*	Ławy k/Birczy	N 49°42ʹ49.56ʹʹ E 22°32ʹ3.14ʹʹ
15	*Rubus bifronus*	Honie	N 50°0ʹ19.28ʹʹ E 22°10ʹ22.06ʹʹ
16	*Rubus perrobustus*	Łazy k/Birczy	N 49°42ʹ49.56ʹʹ E 22°32ʹ3.14ʹʹ
17	*Rubus parthenocisus*	Berendowice	N 49°40ʹ26.44ʹʹ E 22°43ʹ6.76ʹʹ
18	*Rubus pseudidaeus*	Białobrzeszki	N 50°7ʹ18.26ʹʹ E 22°31ʹ29.98ʹʹ
19	*Rubus constrictus*	Berendowice	N 49°40ʹ14.85ʹʹ E 22°43ʹ39.58ʹʹ
20	*Rubus chaerophylloides*	Gruszowa	N 49°40ʹ27.7ʹʹ E 22°41ʹ36.99ʹʹ
21	*Rubus wimmerianus*	Zmysłówka	N 50°9ʹ58.91ʹʹ E 22°22ʹ43.39ʹʹ
22	*Rubus crispomarginatus*	Łazy k/Birczy	N 49°42ʹ49.56ʹʹ E 22°32ʹ3.14ʹʹ
23	*Rubus orthostachys*	Berendowice	N 49°40ʹ14.85ʹʹ E 22°43ʹ39.58ʹʹ

### 2.2. Analysis of the Extracted Amounts of Phenolic Compounds Using Pressure Liquid Extraction and Ultrasonic-Assisted Extraction Methods

**Figure 2:**
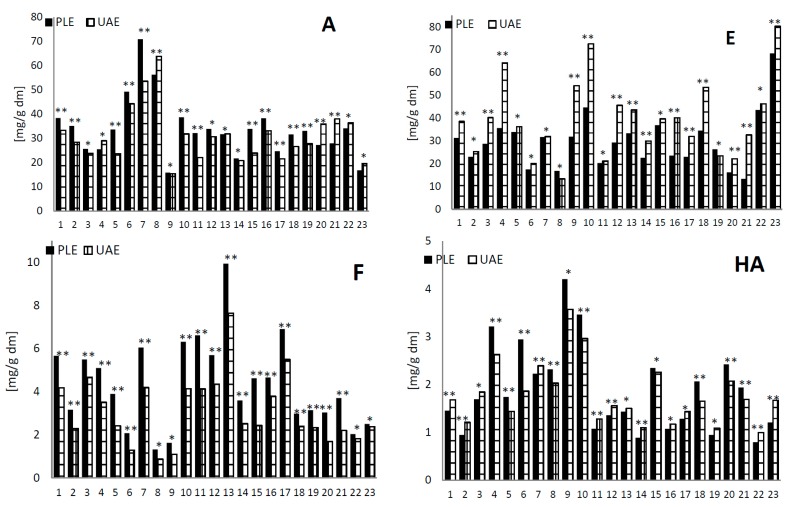
Comparison of the extracted amounts (mg/g dry matter (dm)) of anthocyanins (A), ellagitannins (E), flavonols (F) and hydroxycinnamic acids (HA) of 23 samples of blackberry fruit extracts using pressure liquid extractor (PLE) and ultrasonic-assisted extraction (UAE) methods. Samples followed by the ** were statistically different according to Tukey’s multiple range test (*p* < 0.05); samples followed by the * were not statistically different according to Tukey’s multiple range test (*p* < 0.05).

Analysis of the extracted amounts by PLE and UAE of phenolic compounds of 23 samples is presented in Figure 2. All identified compounds were evaluated; however, the amount was at different levels. The total of flavonols, anthocyanins, hydroxycinnamic acids and ellagitannins was calculated as the sum of compounds resulting from UPLC-PDA/MS analysis. In samples where PLE was applied to the extraction, increased yields of flavonols of 5% to 44% were observed, in comparison to those using UAE. A similar effect was observed by Richter *et al.* [15].

In the case of the extraction of anthocyanins, a slight increase in yields was observed (max. 29% in Sample 5) only in sixteen samples, using the PLE method compared to the UAE. Anthocyanins are more sensitive to thermal degradation than flavonols. Furthermore, Ju *et al.* [32] showed that the PLE system was effective at extracting anthocyanins from grape skins. Anthocyanins are easily oxidized at high temperature, so it is very important to prove that they will not degrade under the proposed PLE conditions [33].

In the analysis of hydroxycinnamic acid extracts, there were no significant differences in the results related to the use of extraction method, PLE or UAE. Slightly more of these compounds was recorded after use of the extraction technique PLE in ten samples and in thirteen after UAE extraction (Figure 2).

For the analysis of ellagic acid and ellagitannins in blackberry fruits, it was established that UAE was a much better method than PLE for preparing extracts. In twenty one samples, more of these compounds (max. 59%) were recorded after use of the UAE extraction technique than PLE (Figure 2). It can be concluded that for the high efficiency extraction of these compounds, the ultrasonic technique works better than PLE. Ellagic acid is a compound that is not easily soluble, so longer contact of the sample with the solvent during extraction by the UAE method probably gives a better effect than the elevated pressure and temperature in the PLE technique. In the case of ellagitannins, better extraction by the UAE method may be due to the release of these compounds from the wall polysaccharides as an effect of the high energy produced by ultrasonic agitation.

### 2.3. Analysis of the Antioxidant Activity Using Pressure Liquid Extraction and Ultrasonic-Assisted Extraction Methods

**Figure 3:**
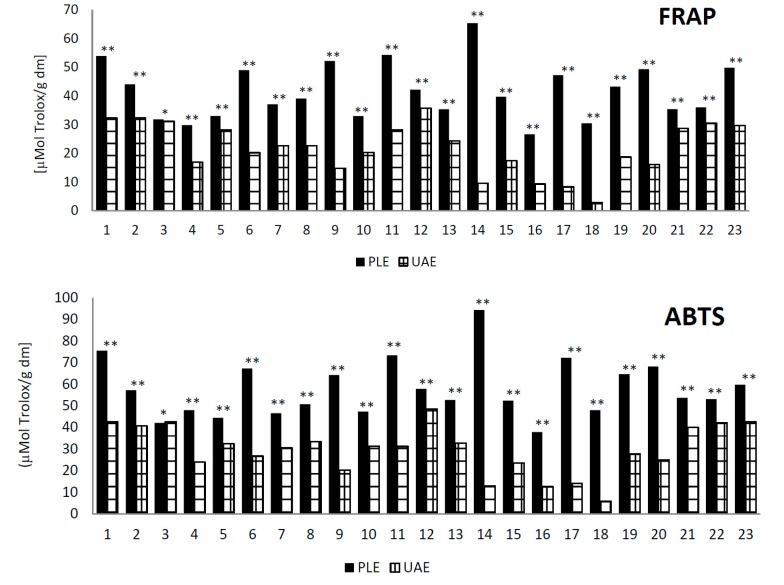
Comparison of the antioxidant activity (µmol Trolox/g dm) of 23 samples of blackberry fruit extracts using PLE and UAE methods. Samples followed by the ** were statistically different according to Tukey’s multiple range test (*p* < 0.05); samples followed by the * were not statistically different according to Tukey’s multiple range test (*p* < 0.05).

Analysis of the antioxidant activity of wild blackberry fruit extracts, measured by the ferric reducing ability of plasma (FRAP) and 2,2-azinobis (3-ethyl-benzothiazoline-6-sulfonic acid) (ABTS) methods, is presented in Figure 3.

In all samples, measuring antioxidant activity by the FRAP and in twenty-two samples as measured by the ABTS method, significantly higher values in the blackberry fruit extracts were obtained by the PLE method rather than the UAE method. In some samples, such as Sample 18, the FRAP antioxidant activity was more than eleven-times greater after extraction by the PLE method rather than the UAE method. Similarly, in the same sample of ABTS, radical scavenging was more than eight-times higher for the PLE technique than for UAE.

### 2.4. Principal Component Analysis

**Figure 4:**
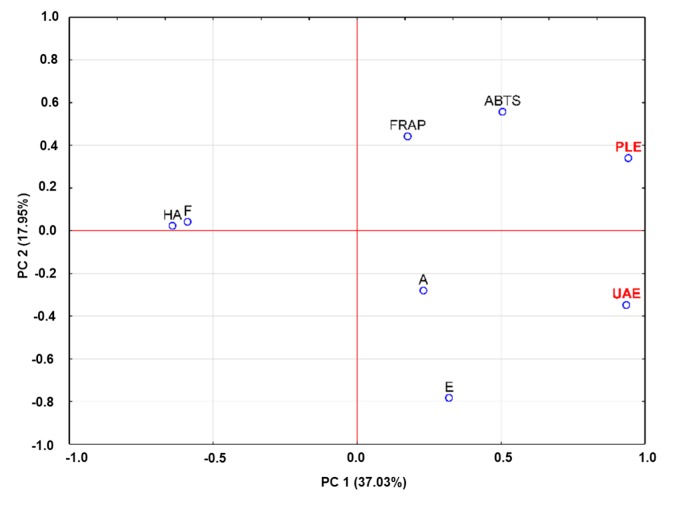
Loading plot for the principal component analysis (PCA) of the first two factors.

Principal component analysis (PCA) was conducted to confirm any relationships among the analyzed variables from the blackberry samples. PCA modeling is good at explaining differences between observations with common variances. After the statistical analysis of all data, the PCA model retained two principal components (PC), which explained 54.99% of the total variability. The loading plots of the first two principal components are shown in Figure 4.

Principal Component 1 (PC1) correlated positively with anthocyanins and ellagic acid; moreover, PC1 was inversely correlated with flavonols and hydroxycinnamic acids. Principal Component 2 (PC2) had high component loadings from the variables analyzed by ABTS and FRAP, whereas it had negative loadings from anthocyanins and ellagic acid and weaker ones with hydroxycinnamic acids and flavonols.

PCA showed that samples of blueberry fruits extracted by the UAE method were characterized by a higher content of anthocyanins (A) and ellagic acid (E) than samples extracted by the PLE method. However, the PLE method produced a relatively good relationship of phenolic compounds to antioxidant activity, as measured by the ABTS and FRAP methods. These results corresponded to the results described above in the section on the influence of extraction methods on the amounts of phenolic compounds.

## 3. Experimental Section

### 3.1. Reagent and Standard

Formic acid and methanol were purchased from Sigma-Aldrich (Steinheim, Germany). Acetonitrile was purchased from Merck (Darmstadt, Germany). Quercetin-3-*O*-glucoside and -galactoside, kaempferol-3-*O*-glucoside, chlorogenic acid, *p*-coumaric acid, cyanidin-3-*O*-glucoside, diglucoside, xyloside and -rutinoside were purchased from Extrasynthese (Lyon, France). Agrimonin (purified 99.98%) was prepared by Bogusław Król from Łódź Polytechnic.

### 3.2. Plant Material

Twenty three different wild blackberry fruit samples were harvested at optimum ripeness in September and October 2013 from various localities throughout southeastern Poland (Table 2). Fruits were directly frozen in liquid nitrogen and freeze-dried (24 h; Alpha 1–4 LSC, Martin Christ GmbH, Osterode am Harz, Germany). Homogeneous powders were obtained by crushing the dried tissues using a closed laboratory mill to avoid hydration (IKA 11A; BIOSAN, Vilno, Lituania). Powders were kept in a refrigerator (−70 °C; Frilabo; Lyon, France) until extract preparation.

### 3.3. Extraction Procedure

#### 3.3.1. Pressurized Liquid Extraction

The Speed Extractor E-916 (BUCHI Labortechnik AG, Flawil, Switzerland) and the procedure of [34] was used for pressurized solvent extraction. Blackberry fruit powders (0.3 g) were mixed with 1 g of diatomaceous earth and placed into 10-mL extraction cells containing a cellulose paper filter at the bottom of each cell. The cells containing the samples were placed into the accelerated solvent system (ASE system), pre-filled with extraction solvent, pressurized and then heated. The extraction conditions and process were as follows: firstly, a static time of 5 min, followed by a flush elution with a 60% volume, followed by a nitrogen purge of 60 s, and then, the samples were extracted twice. The extraction was conducted under the following conditions: solvent: 50% methanol acidified with 1% acetic acid; extraction volume: 25 mL; temperature: 50 °C; pressure: 100 bar. As a result, six samples were processed in one run in exactly the same conditions. Extraction was repeated five times. The diluted extracts were filtered through a hydrophilic politetrafluoroetylen (PTFE) 0.20-µm membrane (Millex Samplicity Filter, Merck, Darmstadt, Germany) and then subjected to UPLC-PDA–MS analysis.

#### 3.3.2. Ultrasound-Assisted Extraction

The powder samples (0.5 g) were extracted with 25 mL of 50% methanol acidified with 1% acetic acid. The extraction was performed twice by incubation for 20 min under sonication (Sonic 6D; Polsonic, Warsaw, Poland). Next, the slurry was centrifuged at 19,000× *g* for 10 min, and the supernatant was filtered through a hydrophilic PTFE 0.20-μm membrane (Millex Samplicity Filter, Merck), diluted and used for analysis. Extraction was repeated five times. The content of polyphenols in individual extracts was determined by means of UPLC-PDA/MS method.

### 3.4. Identification and Quantification of Polyphenols by the Ultra-Performance Liquid Chromatography–Mass Spectrometry Method

Identification of polyphenols in the extracts was carried out using an ACQUITY Ultra Performance LC™ system (UPLC™) with binary solvent manager (Waters Corporation, Milford, MA, USA) and a Micromass Q-Tof Micro mass spectrometer (Waters, Manchester, UK) equipped with an electrospray ionization (ESI) source operating in negative and positive mode. For instrument control, data acquisition and processing, MassLynx™ software (Version 4.1; Waters Corporation, Milford, MA, USA) was used. Separations of individual polyphenols were carried out using a UPLC BEH C18 column (1.7 μm, 2.1 × 100 mm, Waters Corporation) at 30 °C. Samples (10 μL) were injected and elution completed in 15 min with a sequence of linear gradients and isocratic flow rates of 0.45 mL·min^−1^. The mobile phase was composed of Solvent A (4.5% formic acid, *v*/*v*) and Solvent B (100% of acetonitrile). The program began with isocratic elution with 99% A (0 to 1 min); a linear gradient was used until 12 min, lowering A to 25%; from 13.5 to 15.0 min, it was returned to the initial composition (99% A) and then held constant to re-equilibrate the column. Analysis was carried out using full scan, data-dependent MS scanning from *m*/*z* 100 to 1500. The mass tolerance was 0.001 Dalton, and the resolution was 5000. Leucine enkephalin was used as the internal reference compound during ESI-MS accurate mass experiments and was permanently introduced via the LockSpray channel using a Hamilton pump. The lock mass correction was ±1000 for the mass window. All TOF-MS-chromatograms are displayed as base peak intensity (BPI) chromatograms and scaled to 12,400 counts per second (cps) (=100%). The effluent was led directly to an electrospray source with a source block temperature of 130 °C, a desolvation temperature of 350 °C, a capillary voltage of 2.5 kV and a cone voltage of 30 V. Nitrogen was used as the desolvation gas, with a flow rate of 300 L·h^−1^.

Ellagitannins, hydroxycinnamic acids and flavonols were optimized to their estimated molecular mass [M − H]^−^ in the negative mode before and after fragmentation and for anthocyanidin compounds optimized to their estimated molecular mass [M + H]^+^ in the positive mode. The data obtained from UPLC/MS were subsequently entered into the MassLynx 4.0 ChromaLynx™ Application Manager software (Waters Corporation, Milford, MA, USA). Based on these data, the software is able to scan different samples for the characterized substances.

The runs of polyphenolic compounds were monitored at the following wavelengths: ellagitannins at 240 nm, hydroxycinnamates at 320 nm, flavonol glycosides at 360 nm and anthocyanins at 520 nm. The characterization of the single components was carried out via retention time (R_t_), spectra, accurate molecular masses, literature data and pure standards, if available. Calibration curves at concentrations ranging from 0.05 to 5 mg/mL (*r*^2^ ≤ 0.9998) were made for chlorogenic acid, *p*-coumaric acid, quercetin-3-*O*-glucoside and -galactoside, cyanidin-3-*O*-glucoside, -diglucoside, -xyloside and -rutinoside, agrimonin and kaempferol-3-*O*-glucoside as standards. The results were expressed as milligrams per g of dry matter (dm).

### 3.5. Analysis of Antioxidant Activity

The antioxidant activity measured by ABTS^+^ was determined according to the method of Re *et al.* [34]. The total antioxidant potential of the sample was determined using a ferric reducing ability of plasma (FRAP) assay by Benzie *et al.* [35] as a measure of antioxidant power. A standard curve was prepared for all analyses, using different concentrations of Trolox. All determinations were performed in triplicate using a Shimadzu UV-2401 PC spectrophotometer (Kyoto, Japan). The results were corrected for dilution and expressed in micromoles of Trolox per gram dm.

### 3.6. Statistical Analysis

One-way analysis of variance (ANOVA) was used for comparison of the results. The method used to discriminate among the means was Tukey’s procedure. Significance was defined at *p* ≤ 0.05. Principal component analysis (PCA) was applied to the mean values of the measured traits. Analyses were performed using Statistica Version 10 (StatSoft, Krakow, Poland).

## 4. Conclusions

PLE may be a more suitable method for quantitative analysis of heat-stable components, such as flavonols, while the UAE method may be applicable to the preparation of heat-labile hydroxycinnamic acid extracts. PLE is automatic, as well as performed in an inert atmosphere and protected from light, which decreases the risk of the formation of free radicals during sample preparation for antioxidant activity measurement. Additionally, these new techniques enable faster extraction, in which a smaller amount of solvents is used and higher yields are obtained in comparison with traditional solvent extraction. Therefore, it would be appropriate to optimize the conditions and solvents used for extraction of polyphenolic substances from plant sources.
